# Non-Contrasted CT Radiomics for SAH Prognosis Prediction

**DOI:** 10.3390/bioengineering10080967

**Published:** 2023-08-16

**Authors:** Dezhi Shan, Junjie Wang, Peng Qi, Jun Lu, Daming Wang

**Affiliations:** 1 Department of Neurosurgery, Beijing Hospital, National Center of Gerontology, Institute of Geriatric Medicine, Chinese Academy of Medical Sciences, Beijing 100730, China; doctorshann@163.com (D.S.);; 2 Graduate School, Peking Union Medical College, Beijing 100730, China

**Keywords:** subarachnoid hemorrhage, non-contrast CT, radiomics, prognosis, machine learning algorithms

## Abstract

Subarachnoid hemorrhage (SAH) denotes a serious type of hemorrhagic stroke that often leads to a poor prognosis and poses a significant socioeconomic burden. Timely assessment of the prognosis of SAH patients is of paramount clinical importance for medical decision making. Currently, clinical prognosis evaluation heavily relies on patients’ clinical information, which suffers from limited accuracy. Non-contrast computed tomography (NCCT) is the primary diagnostic tool for SAH. Radiomics, an emerging technology, involves extracting quantitative radiomics features from medical images to serve as diagnostic markers. However, there is a scarcity of studies exploring the prognostic prediction of SAH using NCCT radiomics features. The objective of this study is to utilize machine learning (ML) algorithms that leverage NCCT radiomics features for the prognostic prediction of SAH. Retrospectively, we collected NCCT and clinical data of SAH patients treated at Beijing Hospital between May 2012 and November 2022. The modified Rankin Scale (mRS) was utilized to assess the prognosis of patients with SAH at the 3-month mark after the SAH event. Based on follow-up data, patients were classified into two groups: good outcome (mRS ≤ 2) and poor outcome (mRS > 2) groups. The region of interest in NCCT images was delineated using 3D Slicer software, and radiomic features were extracted. The most stable and significant radiomic features were identified using the intraclass correlation coefficient, *t*-test, and least absolute shrinkage and selection operator (LASSO) regression. The data were randomly divided into training and testing cohorts in a 7:3 ratio. Various ML algorithms were utilized to construct predictive models, encompassing logistic regression (LR), support vector machine (SVM), random forest (RF), light gradient boosting machine (LGBM), adaptive boosting (AdaBoost), extreme gradient boosting (XGBoost), and multi-layer perceptron (MLP). Seven prediction models based on radiomic features related to the outcome of SAH patients were constructed using the training cohort. Internal validation was performed using five-fold cross-validation in the entire training cohort. The receiver operating characteristic curve, accuracy, precision, recall, and f-1 score evaluation metrics were employed to assess the performance of the classifier in the overall dataset. Furthermore, decision curve analysis was conducted to evaluate model effectiveness. The study included 105 SAH patients. A comprehensive set of 1316 radiomics characteristics were initially derived, from which 13 distinct features were chosen for the construction of the ML model. Significant differences in age were observed between patients with good and poor outcomes. Among the seven constructed models, model_SVM exhibited optimal outcomes during a five-fold cross-validation assessment, with an average area under the curve (AUC) of 0.98 (standard deviation: 0.01) and 0.88 (standard deviation: 0.08) on the training and testing cohorts, respectively. In the overall dataset, model_SVM achieved an accuracy, precision, recall, f-1 score, and AUC of 0.88, 0.84, 0.87, 0.84, and 0.82, respectively, in the testing cohort. Radiomics features associated with the outcome of SAH patients were successfully obtained, and seven ML models were constructed. Model_SVM exhibited the best predictive performance. The radiomics model has the potential to provide guidance for SAH prognosis prediction and treatment guidance.

## 1. Introduction

Subarachnoid hemorrhage (SAH), the third most prevalent subtype of stroke, is a critical condition that poses a significant threat to patients’ lives and imposes a substantial social and economic burden [1,2]. Proper and timely prognosis prediction holds paramount significance in the case of patients afflicted with SAH, playing a pivotal role in guiding judicious treatment determinations. This process not only facilitates precise risk quantification but also aids in the discernment of the most fitting therapeutic strategies and the formulation of meticulously tailored care blueprints. Additionally, prognostic assessment offers precise and comprehensive information to both patients and their families regarding the gravity of the disease, possible clinical trajectories, and the imperative nature of therapeutic interventions. This multifaceted approach enhances collaborative engagement in the comprehensive oversight of the patient’s medical condition. Such an in-depth prognostic evaluation engenders an atmosphere of informed partnership, thereby augmenting the effective orchestration of the treatment regimen.

Currently, non-contrast computed tomography (NCCT) scans stand as the foremost diagnostic modality for SAH [3]. However, the conventional clinical interpretation of NCCT images, while valuable, offers a limited scope of informative insights. In this context, the burgeoning field of radiomics, an evolving imaging technology, has garnered attention. Radiomics entails the extraction of quantitative features from medical images, serving as diagnostic markers of paramount significance [4,5,6]. This approach presents distinct merits, notably its non-invasive nature and cost-effectiveness, both of which underpin its capacity to establish the bedrock for personalized medical interventions. Consequently, radiomics assumes an increasingly indispensable role in the panorama of disease diagnosis and prognosis prediction. With its foundations deeply rooted in the field of artificial intelligence, the paradigm of machine learning (ML) has pervaded an array of diverse facets within our daily routines [7,8,9,10]. ML algorithms, emerging as indispensable tools, play a pivotal role in facilitating precise diagnostics and comprehensive treatments of conditions such as tumors, cardiovascular ailments, and cerebrovascular diseases [11,12,13]. Through the harnessing of data-derived insights, the application of ML techniques is actively reshaping the contemporary landscape of medical practice, exemplifying a significant transformation in the field.

Previous studies have primarily focused on the prognosis of SAH based on clinical information and imaging characteristics [14,15,16,17]. Additionally, some investigations have explored the prediction of hemorrhage expansion and functional outcome assessment of spontaneous intracerebral hemorrhage through the utilization of radiomics features obtained from NCCT [18,19,20,21,22]. However, there are few studies on the prognosis prediction of SAH based on NCCT radiomics features using ML algorithms. The fundamental objective of this study was to employ ML algorithms with the purpose of accurately predicting the prognosis of SAH, utilizing radiomics features extracted from NCCT scans. Through the construction of a predictive model using ML algorithms, the study aimed to provide valuable insights for guiding prognostic assessments and treatment strategies in SAH cases.

## 2. Results

### 2.1. Clinical Features

The study included a total of 105 patients diagnosed with SAH, with an average age of 59.64 ± 11.29 years. Within this participant pool, the male demographic comprised 39 individuals, representing a proportion of 37.14%. In terms of other pertinent metrics, a total of 26 participants, accounting for 24.76%, had a history of smoking. Similarly, an equivalent proportion of 24.76% (26 participants) had a history of alcohol consumption. Among the broader participant demographic, a notable 62 individuals (59.05%) presented a medical history of hypertension (HTN), while 16 individuals (15.24%) had a documented history of diabetes mellitus (DM).

Out of these, 85 patients were categorized as having a good outcome, while 20 patients experienced a poor outcome. The only notable distinction between the two groups was identified in terms of age. No significant differences were observed in terms of sex, smoking history, alcohol history, history of HTN, or history of DM between the group with good outcomes and the group with poor outcomes. Detailed clinical characteristics are displayed in Table 1.

### 2.2. Stable Feature Selection

**Figure 1:**
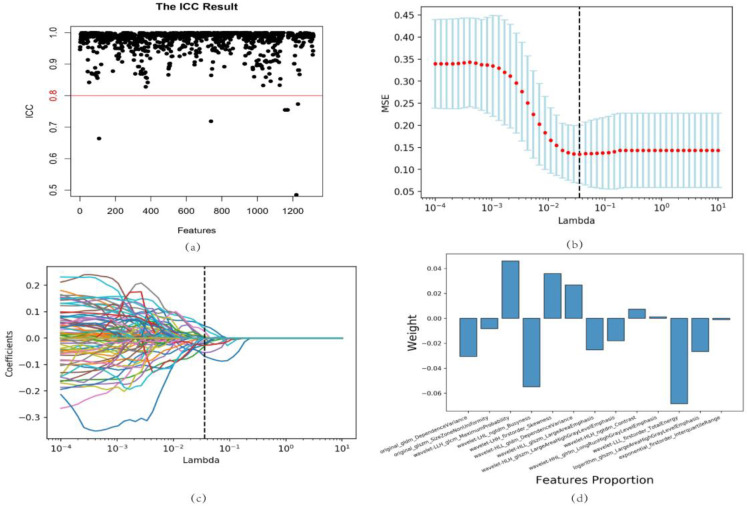
Feature selection and proportion of radiomics features. (**a**) ICC test results. The features with an ICC > 0.8 were selected for further research. (**b**) Five-fold cross-validation for radiomics features selection in the LASSO regression model. The black vertical line represents the minimum error. (**c**) LASSO regression coefficient profiles. Colored lines depict features and illustrate how their coefficients change with different regularization levels. (**d**) Radiomics feature coefficients based on LASSO regression results. ICC, intraclass correlation coefficient; LASSO, least absolute shrinkage and selection operator.

In the preliminary analysis, a total of 1316 radiomics features were derived from the NCCT scans, encompassing a spectrum of attributes, including first-order features, shape features, and texture features. After data processing and intraclass correlation coefficient (ICC) assessment, 1266 radiomics features remained for further analysis. A *t*-test and least absolute shrinkage and selection operator (LASSO) regression were performed, resulting in the selection of 13 radiomics features that significantly differed between the different outcome groups. These features were ‘original_gldm_DependenceVariance’, ‘original_glszm_SizeZoneNonUniformity’, ‘wavelet-LLH_glcm_MaximumProbability’, ‘wavelet-LHL_ngtdm_Busyness’, ‘wavelet-LHH_firstorder_Skewness’, ‘wavelet-HLL_gldm_DependenceVariance’, ‘wavelet-HLL_glszm_LargeAreaEmphasis’, ‘wavelet-HLH_glszm_LargeAreaHighGrayLevelEmphasis’, ‘wavelet-HLH_ngtdm_Contrast’, ‘wavelet-HHL_glrlm_LongRunHighGrayLevelEmphasis’, ‘wavelet-LLL_firstorder_TotalEnergy’, ‘logarithm_glszm_LargeAreaHighGrayLevelEmphasis’, and ‘exponential_firstorder_InterquartileRange’ (Figure 1).

### 2.3. Predictive Model Construction and Evaluation

Various ML algorithms were utilized to construct the model, encompassing logistic regression (LR), support vector machine (SVM), random forest (RF), light gradient boosting machine (LGBM), adaptive boosting (AdaBoost), extreme gradient boosting (XGBoost), and multi-layer perceptron (MLP). A series of seven ML predictive models were generated, denoted as follows: model_LR, model_SVM, model_RF, model_LGBM, model_AdaBoost, model_XGBoost, and model_MLP. These models were rigorously evaluated using 5-fold cross-validation. Among the models, the model_SVM demonstrated the most impressive performance. During cross-validation on the training cohort, it achieved an average area under the curve (AUC) of 0.98 (standard deviation: 0.01), indicating excellent predictive accuracy. The robustness of the model was further confirmed in the testing cohort, where it maintained a high level of performance with an average AUC of 0.88 (standard deviation: 0.08). The utilization of 5-fold cross-validation ensures the reliable and unbiased estimation of model performance. These cross-validated AUC values reinforce the reliability and generalizability of the model_SVM in accurately predicting outcomes.

**Figure 2:**
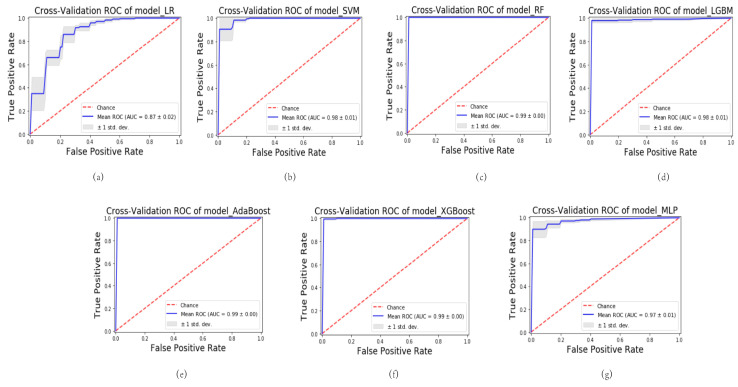
Results of cross-validation of different models in the training cohort. (**a**) Cross-validation of model_LR. (**b**) Cross-validation of model_SVM. (**c**) Cross-validation of model_RF. (**d**) Cross-validation of model_LGBM. (**e**) Cross-validation of model_AdaBoost. (**f**) Cross-validation of model_XGBoost. (**g**) Cross-validation of model_MLP. LR, logistic regression; SVM, support vector machine; RF, random forest; LGBM, light gradient boosting machine; AdaBoost, adaptive boosting; XGBoost, extreme gradient boosting; MLP, multi-layer perception.

**Figure 3:**
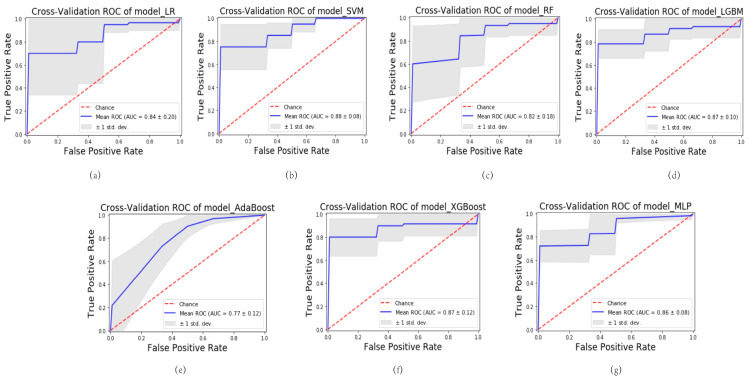
Results of cross-validation of different models in the testing cohort. (**a**) Cross-validation of model_LR. (**b**) Cross-validation of model_SVM. (**c**) Cross-validation of model_RF. (**d**) Cross-validation of model_LGBM. (**e**) Cross-validation of model_AdaBoost. (**f**) Cross-validation of model_XGBoost. (**g**) Cross-validation of model_MLP.

The results illustrated in Figure 2 and Figure 3 demonstrate varying levels of performance among the other models. In the training cohort, these models exhibited a spectrum of average AUC values, ranging from 0.87 to 0.99. Similarly, in the testing cohort, the AUC values ranged from 0.77 to 0.87. This highlights the differences in predictive accuracy achieved by each model.

**Figure 4:**
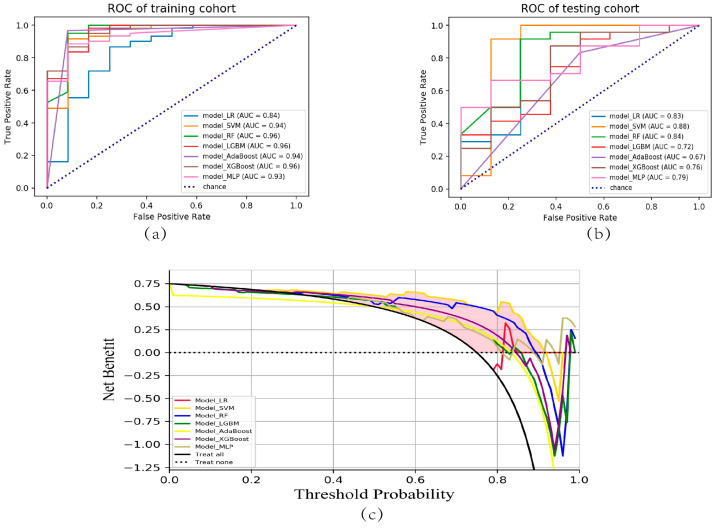
ROC and DCA curves of the seven ML models. (**a**) ROC curves for model_LR, model_SVM, model_RF, model_LGBM, model_AdaBoost, model_XGBoost, and model_MLP within the training cohort are presented. (**b**) The corresponding ROC curves for the testing cohort encompassed the same set of models. (**c**) Additionally, the DCA curves were constructed for these models in the testing cohort. ROC, receiver operating characteristic; DCA, decision curve analysis; ML, machine learning.

The receiver operating characteristic (ROC) curves for all models, encompassing both the training and testing cohorts, have been visualized in Figure 4a,b. The model_SVM demonstrated a high level of performance in the training cohort, achieving an AUC value of 0.94, indicating excellent predictive accuracy. Notably, in the testing cohort, the model_SVM outperformed all other models, exhibiting an AUC value of 0.88. These findings highlight the superior predictive capability of the model_SVM, suggesting its potential as a robust and reliable tool for accurate outcome prediction. The decision curve analysis (DCA) curve indicated that the RF model had the highest net benefit among the different models (Figure 4c).

The evaluation results for precision, recall, accuracy, and the f1-score of each model in the testing cohort are exhaustively delineated in Table 2. In summary, the model_SVM model utilizing the selected radiomics features exhibited the highest performance in distinguishing between different prognostic outcomes among SAH patients. It demonstrated exceptional accuracy and achieved high AUC values, underscoring its potential for clinical application in the prognostic stratification of SAH patients.

## 3. Discussion

SAH is a severe form of hemorrhagic stroke that carries a significant socioeconomic burden and often leads to poor prognosis. Assessing the prognosis of patients diagnosed with SAH holds utmost significance in guiding clinical decisions pertaining to both diagnosis and treatment strategies for SAH [15,23]. By accurately predicting a patient’s prognosis, clinicians can effectively evaluate preoperative risks, opt for the most appropriate therapeutic approaches, and formulate individualized treatment regimens. The comprehensive prognostic assessment serves an additional purpose in furnishing precise insights into the disease’s severity, potential ramifications, and the imperative of timely intervention for patients and their families, thereby fostering an enhanced milieu of collaborative treatment engagement. This multifaceted prognostic evaluation, thus, assumes a pivotal role not only in clinical management but also in patient communication and shared decision-making.

NCCT scans of the brain have demonstrated high efficacy in the detection of high-density hemorrhagic foci, rendering them a pivotal modality in clinical practice for the diagnosis of SAH due to their rapid acquisition and cost-effectiveness. Radiomics, a data-extraction approach characterized by its ability to automatically derive an extensive spectrum of quantitative imaging features, has emerged as a compelling frontier within the realm of medical imaging. This computational technique holds exceptional promise, offering the potential to furnish clinicians with supplementary, non-invasive insights. These insights, in turn, stand to substantially enhance the precision and confidence of clinical decision-making processes. Despite this potential, there are limited radiomics models available for predicting the prognosis of SAH. Within the scope of this research, radiomics characteristics were extracted from NCCT scans, followed by the development of ML models aimed at predicting the prognosis of SAH.

The comparative analysis of clinical data derived from patients exhibiting good and poor outcomes has unveiled a noteworthy dissimilarity in terms of age distribution between the two cohorts. Aging stands as a widely acknowledged and pivotal risk determinant linked to a less favorable prognosis among individuals afflicted with SAH, as substantiated by previous studies [24,25]. This discovery holds noteworthy implications for clinical practice. It emphasizes the need for increased attention and tailored interventions when treating this group of patients. Understanding the impact of advanced age on the progression of SAH becomes crucial for enhancing patient outcomes and guiding treatment strategies. This involves re-evaluating and adjusting strategies to better suit the distinctive requirements of the elderly SAH population. Nevertheless, in terms of various other clinical characteristics, no statistically significant differences were observed between the studied groups. These results suggest that, within the parameters examined, there is a lack of noteworthy divergence among the groups in relation to these clinical features. During the extraction and selection of radiomics features, 13 features were found to be significantly different between the SAH patients with different prognoses, including 3 first-order features, and 10 texture features. The most important feature based on the coefficients from LASSO regression was ‘wavelet-LLL_firstorder_TotalEnergy’, ‘wavelet-LHL_ngtdm_Busyness’, and ‘wavelet-LLH_glcm_MaximumProbability’. Wavelet filtering assumes a critical role in the extraction of imaging biomarkers, effectively augmenting the representational capacity and classification performance of these features [26,27]. Within the framework of this study, pronounced distinctions were observed among patients with SAHs of diverse prognoses regarding the imaging biomarkers extracted via wavelet filtering.

Enhancements in computational capabilities and algorithmic refinements have propelled the substantial headway of ML in prognosticating ailments [28,29,30]. In this study, seven predictive models were constructed to differentiate between SAH patients with different prognoses, with model_SVM demonstrating the best performance. The SVM has emerged as a prominent supervised learning algorithm that finds extensive application in the domains of pattern recognition and ML. Owing to its remarkable characteristics, this model demonstrates a superior ability to generalize across diverse datasets. Notably, the SVM adeptly accommodates situations involving limited sample sizes and high-dimensional data, thereby rendering it highly suitable for intricate feature-based predicaments. The utility of SVM becomes particularly evident in scenarios where the underlying patterns are intricate and necessitate sophisticated computational methodologies for accurate analysis and prediction. SVM finds extensive applications in disease diagnosis, prognosis assessment, and biomarker research [31,32,33,34]. Model_SVM effectively distinguished SAH patients with different prognoses in both the training and testing cohorts.

Based on the internal validation through cross-validation, the model_SVM demonstrated exceptional performance, suggesting its robustness and accuracy. It outperformed the other six models in terms of AUC value, accuracy, precision, sensitivity, and f1 score in the testing cohort, indicating better discriminatory power. Furthermore, the DCA revealed that model_SVM provided the highest net benefit. This suggests that using model_SVM for clinical decision-making has excellent clinical applicability, as it offers greater benefit compared to the other models evaluated. Overall, based on these evaluations and analyses, model_SVM stands out as a highly reliable and accurate predictive model with a superior performance and strong clinical applicability.

Currently, the prognosis prediction of SAH patients relies on the clinical experience of physicians and radiologists, which can introduce bias and errors. Accurately predicting the prognosis of SAH patients holds paramount significance in guiding treatment strategies and making informed decisions. Among the ML models formulated within this investigation, particularly model_SVM demonstrates a remarkable aptitude for precisely foretelling the prognostic landscape of SAH patients, providing essential information for determining appropriate treatment strategies. The integration of ML-based prediction models incorporating NCCT radiomics features carries profound clinical implications for SAH prognosis. These computational models play a pivotal role across multiple dimensions. This methodology not only facilitates a more accurate assessment of patient risks, but also offers invaluable insights for selecting optimal therapeutic strategies. Moreover, these models provide indispensable support in the realm of personalized treatment planning, underscoring their significance within the broader spectrum of medical care. This holistic and comprehensive approach has the transformative potential to reshape both SAH diagnosis and treatment, resulting in a tangible enhancement of patient outcomes.

However, it is essential to acknowledge the limitations of this study. Firstly, the retrospective design of the study might introduce selection bias, potentially compromising the generalizability of the results. Moreover, the investigation was executed within a solitary institution, encompassing a sample size that is relatively modest. This circumstance could potentially influence the statistical robustness and the broader applicability of the radiomics model. Additionally, the exploration was bound by practical constraints that precluded the execution of a multicenter data validation process. Therefore, it remains imperative to meticulously validate and refine the model further, ensuring its steadfastness and utility across a diverse range of clinical settings. Subsequent investigations should give precedence to the incorporation of more extensive and heterogeneous cohorts from multiple centers, thereby reinforcing the dependability and applicability of findings. In upcoming research endeavors, the incorporation of images characterized by thinner slice thickness holds comparable significance. This augmentation in imaging data is likely to provide a more comprehensive delineation of latent features and patterns, leading to increased accuracy and robustness within the model. This endeavor would foster a more exhaustive comprehension of the performance and potential clinical utility intrinsic to the ML-based predictive model. The strategic resolution of these limitations is poised to contribute significantly to the advancement and wider dissemination of radiomics-based prognosis prediction for SAH. While the clinical translation of ML-based radiomics models necessitates sustained development, the advancement of artificial intelligence is progressively propelling the astute metamorphosis of clinical diagnosis and treatment paradigms. Notwithstanding the intrinsic limitations inherent to the present study, it is anticipated that its findings will duly serve as a wellspring of inspiration for forthcoming investigations, thereby furnishing invaluable guidance to inform and shape clinical practices. With the inclusion of more diverse populations and datasets, its clinical guiding value will continue to be enhanced.

## 4. Materials and Methods

### 4.1. Study Population

**Figure 5:**
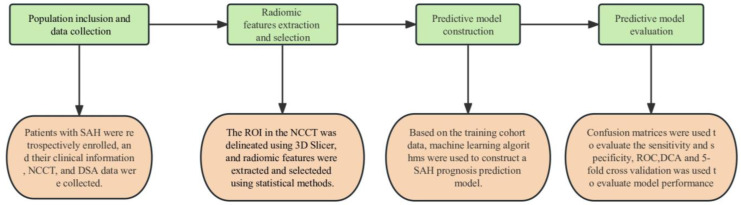
The study workflow. The study comprises four parts: population inclusion and data collection, radiomic features extraction and selection, predictive model construction, and predictive model evaluation.

The study population consisted of patients with SAH who were treated at Beijing Hospital between May 2012 and October 2022. The study enrolled adult patients, aged 18 years or older, who were diagnosed with a SAH based on NCCT. Furthermore, these patients were subjected to follow-up assessments after a period of 3 months. Patients without available clinical records, NCCT images of poor quality, and multiple scans were excluded from the study. The study workflow is shown in Figure 5.

### 4.2. Clinical and Imaging Data

Clinical and NCCT data were collected retrospectively. The clinical information encompassed the patient’s gender, age, smoking and alcohol history, as well as their history of HTN and DM. The modified Rankin Scale (mRS) was utilized to assess the prognosis of patients with SAH. Additionally, mRS regarding the follow-up evaluations conducted after a three-month period was collected. Based on the results of mRS, patients were divided into two groups: the good outcome group (mRS 0–2) and the poor outcome group (mRS 3–6). NCCT scans were performed using the Aquilion ONE CT scanner (Canon Medical Systems, Otawara, Japan) with a detector row of 320 × 0.5 mm. The scanning parameters included 135 kV, 300 mAs, and reconstruction with adaptive iterative dose reduction. The reconstructed slice thickness was 5 mm.

### 4.3. Image Segmentation and Feature Extraction

Image segmentation was conducted using 3D Slicer software, with the region of interest (ROI) delineated on the NCCT images to isolate the cerebral hemorrhage area. Radiomic features were extracted using the Pyradiomics package in Python. The extracted features included first-order features, shape features, and texture features.

To select the most relevant radiomics features for the study, the ICC was calculated to assess the stability of the features. Features with an ICC greater than 0.8 were chosen for further analysis. The selected features were normalized, and the homogeneity of variances was tested using the Levene test. A *t*-test was then performed to identify significantly different variables between the good-outcome and poor-outcome groups. Features with a p-value less than 0.05 were considered statistically significant. The LASSO regression was utilized to further refine the selection of the most significant parameters.

### 4.4. Predictive Model Construction and Evaluation

The process of constructing predictive models involved a randomized allocation of the dataset into distinct training and testing cohorts, with a distribution ratio of 7:3. Various ML algorithms were utilized to construct predictive models based on the radiomic features associated with the patients’ recovery outcomes within the training cohort. These algorithms encompassed LR, SVM, RF, LGBM, AdaBoost, XGBoost, and MLP. To evaluate the performance of the predictive models, five-fold cross-validation was conducted in the training cohort. Confusion matrices were used to assess performance, and ROC curves were plotted to determine the AUC as a measure of predictive performance. Accuracy, precision, recall, and f-1 score evaluation metrics were calculated. Additionally, DCA was performed on each model to evaluate its effectiveness.

### 4.5. Statistical Analysis

The statistical analysis was carried out utilizing the R software package. This analysis encompassed the application of the Levene test, chi-square test, *t* test, and ROC analysis. A significance threshold of *p* = 0.05 was employed for statistical evaluations.

## 5. Conclusions

The study successfully identified relevant radiomics features associated with SAH patients’ outcomes and demonstrated the high accuracy of the model_SVM using NCCT radiomics features. These findings suggest that the radiomics model has the potential to enhance prognosis prediction and treatment efficiency for SAH.

## Figures and Tables

**Table 1:** Clinical characteristics.

Variable		Total *n* = 105		*p* Value
		Good Outcome (85)	Poor Outcome (20)	
Sex	Male	31	8	0.77
	Female	54	12	
Age	<60	46	6	0.05
	≥60	39	14	
Hypertension	Yes	50	12	0.92
	No	35	8	
Diabetes Mellitus	Yes	11	5	0.18
	No	74	15	
Smoking	Yes	20	6	0.55
	No	65	14	
Drinking	Yes	22	4	0.58
	No	63	16	

**Table 2:** Performance of ML models.

Model	Accuracy	Precision	Recall	F1-Score
Model_LR	0.75	0.56	0.75	0.64
Model_SVM	0.84	0.87	0.84	0.82
Model_RF	0.78	0.76	0.78	0.74
Model_LGBM	0.81	0.80	0.81	0.80
Model_AdaBoost	0.75	0.75	0.75	0.75
Model_XGBoost	0.84	0.84	0.84	0.83
Model_MLP	0.81	0.85	0.81	0.77

## Data Availability

Not applicable.
